# The Long Harm of Childhood: Childhood Exposure to Mortality and Subsequent Risk of Adult Mortality in Utah and The Netherlands

**DOI:** 10.1007/s10680-018-9505-1

**Published:** 2018-11-28

**Authors:** Ingrid K. van Dijk, Angelique Janssens, Ken R. Smith

**Affiliations:** 1 Department of History, Radboud University Nijmegen, Erasmusplein 1, 6500 HD Nijmegen, The Netherlands; 2 Faculty of Arts and Social Sciences, Maastricht University, Maastricht, The Netherlands; 3 Department of Family and Consumer Studies and Population Sciences, Huntsman Cancer Institute, University of Utah, Salt Lake City, UT USA

**Keywords:** Human aging, Infection, Biodemography, Epidemiology, Early-life adversity, Cause-specific mortality, Exposure to disease, Adult mortality

## Abstract

How do early-life conditions affect adult mortality? Research has yielded mixed evidence about the influence of infant and child mortality in birth cohorts on adult health and mortality. Studies rarely consider the specific role of mortality within the family. We estimated how individuals’ exposure to mortality as a child is related to their adult mortality risk between ages 18 and 85 in two historical populations, Utah (USA) 1874–2015 and Zeeland (The Netherlands) 1812–1957. We examined these associations for early community-level exposure to infant and early (before sixth birthday) and late (before eighteenth birthday) childhood mortality as well as exposure during these ages to sibling deaths. We find that that exposure in childhood to community mortality and sibling deaths increases adult mortality rates. Effects of sibling mortality on adult all-cause mortality risk were stronger in Utah, where sibling deaths were less common in relation to Zeeland. Exposure to sibling death due to infection was related to the surviving siblings’ risk of adult mortality due to cardiovascular disease (relative risk: 1.06) and metabolic disease (relative risk: 1.42), primarily diabetes mellitus, a result consistent with an inflammatory immune response mechanism. We conclude that early-life conditions and exposure to mortality in early life, especially within families of origin, contribute to adult mortality.

## Introduction

The first birth cohorts which experienced decreasing mortality in childhood were also the first to experience an increase in life expectancy in adulthood (Crimmins and Finch 2006), suggesting that health and survival in adulthood may be influenced by early-life exposure to disease and famine (Barker et al. 2002; Bengtsson and Lindström 2000; Finch and Crimmins 2004; Fridlizius 1989). Support for this theory has been mixed, however. Research for rural Sweden showed that high infant and child mortality within birth cohorts is related to lower life expectancies of survivors of these cohorts (Bengtsson and Lindström 2000; Costa 2000; Quaranta 2014) while in other cases no such relationship was found (Cohen et al. 2010; Gagnon and Mazan 2009; Hayward et al. 2016). Evidence linking early-life exposure to infections to old-age mortality is often based on studies using (aggregate) cohort-level data. These studies could therefore represent overall shifts in mortality patterns in the population without demonstrating a causal link between early-life exposures and mortality patterns in adulthood. Moreover, as geographical and familial variation in health and survival exists, individuals born in the same period can experience heterogeneity in their exposure to mortality. Therefore, aggregate measures of exposure to infant and child mortality in large birth cohorts using excess mortality or crude death rates are likely to bias estimates relating exposure to mortality in early life to subsequent adult mortality. Exposure to mortality can be measured more precisely by using community-level measures but especially by focusing on mortality among siblings. In this study, we focus on the relation between childhood exposure to mortality in communities and in families and subsequent adult mortality.

It is axiomatic that early-life conditions are shaped within families. Nonetheless, little consensus exists about the families’ role linking family deaths in childhood and subsequent adult mortality. When research considers the role of families, commonly, it is to account for confounding factors (Öberg 2015). For instance, Gagnon and Mazan (2009) controlled for family-level conditions in research regarding the relation between cohort mortality and old-age mortality and found that family-level characteristics have stronger effects than exposure to cohort-level mortality. However, as they measured mortality exposure through mortality in birth cohorts and not through more proximate exposures in families, exposure to mortality in childhood may, in fact, play a much larger role in adult mortality than previously thought. Other studies have looked at the influence of sibling mortality on the life course and found that sibling deaths affect life transitions such as timing of marriage and fertility (Störmer and Lummaa 2014). In addition, recent research has shown that in modern-day Scandinavia childhood exposure to sibling mortality leads to an increased likelihood of death between birth and age 37 (Yu et al. 2017), suggesting that sibling deaths have a lasting influence on health and mortality; however, it remains unknown whether survival is similarly affected in later adulthood.

With regard to the mechanisms linking early-life mortality exposure to later-life mortality, several mechanisms are relevant here: causal mechanisms, shared environments in childhood and adulthood, and mechanisms of scarring and selection. First, bacterial and viral infections are related to inflammatory immune responses and stunting in adulthood, indicators of adverse health (Finch and Crimmins 2004). Sibling deaths indicate that individuals may have been directly exposed to these infections. Inflammatory immune responses and inadequate development of vital organs and the immune system may lead to reduced life expectancy (Barker et al. 2002; Doblhammer et al. 2013; Lindeboom et al. 2010) through increased risks of stroke, cardiovascular disease (Caruso et al. 2005), metabolic diseases (Crimmins and Finch 2006) and other causes of death in adulthood (Vasto et al. 2007). In addition, sibling mortality may lead to chronically increased levels of stress (Yu et al. 2017), which, in turn, is a risk factor for diseases such as asthma, diabetes, gastrointestinal disorders, cancer and myocardial infection (McEwen 1998; McEwen and Stellar 1993). Similarly, Norton et al. (2017) found that sibling deaths during childhood predict systemic inflammation in later life, linking stress responses to adult life expectancy. To gain further insights into the causal mechanisms linking sibling mortality to mortality hazards in adulthood, in this paper, we employ cause of death information and competing risk models.

Second, individuals who experienced sibling deaths may originate from disadvantaged socioeconomic backgrounds related to poor living and nutritional circumstances, leading to both elevated infant and child mortality and a compromised start in life for survivors. When infants were not consistently breastfed, the use of contaminated water may have led to elevated mortality risks for infants and families and adverse health outcomes in adulthood. Access to nutrition is expected to affect only certain segments of the populations studied here, as the influence of nutrition on child survival decreased during the nineteenth century. Beyond the effects on health in childhood, childhood conditions are related to conditions in adulthood through processes of social reproduction. Life spans of individuals exposed to poor conditions in early life may therefore also be affected by the same adverse conditions in midlife resulting in shortened life expectancy (Preston et al. 1998). Third, high cohort or family infant and child mortality may coexist with relatively favorable adult survival. Within high-mortality cohorts, frail individuals may be selected out, resulting in a relatively robust surviving cohort (Costa 2000; Doblhammer et al. 2013; Myrskylä 2010) or sibship (Alter et al. 2001). In addition, individuals from favorable childhood and adult environments may be spared (Preston et al. 1998). Apart from these selection effects, some exposures may lead to acquired immunity (Preston et al. 1998). Although some research has shown that scarring effects commonly dominate selection effects (Hatton 2011), the extent to which this is true may vary by the strength of exposure to mortality in the population: Less lethal exposures may generate selection effects, while stronger exposures could have direct physiological effects on survivors leading to scarring (Störmer and Willführ 2010). In this paper, we take the degree of exposure into account in order to disentangle selection and scarring effects.

Deaths among siblings may have different effects within the sibling group, as individuals are at different ages when deaths occur (Störmer and Lummaa 2014). There is no consensus in the literature about the age at which exposure to mortality may affect later health outcomes. Famines are related to shorter life expectancies for newborns at the time of the famine (Lindeboom et al. 2010) but also for children who are exposed in utero during mortality peaks (Barker et al. 2002; Bengtsson and Lindström 2003; Doblhammer 2003). Bruckner and Catalano (2009) found no effects of cohort infant mortality on adult mortality rates and hypothesize that the critical period may be at older age. Other researchers hypothesized that exposure to mortality is significant up to age 5 (Fridlizius 1989) or 15 (Crimmins and Finch 2006). Alter et al. (2001) analyzed sibling mortality to assess the role of health behaviors in explaining excess mortality in early and later life. They found that sibling mortality affects mortality among other siblings during childhood but diminishes after age 15 with no effects after age 55, while demonstrating evidence consistent with acquired immunity after age 30. However, the authors used all sibling deaths in the family of origin, omitting the possibility that individuals may not have been born at the time of death of their sibling, or reached an age at which mortality exposure was less relevant for adult survival. In this paper, we use mortality when ego is in utero, between birth and age 5, age 6 and 18, and all mortality between birth and age 18.

In summary, the innovation of this paper is the estimation of adult mortality risks associated with mortality exposure in neighborhoods and communities while simultaneously measuring direct exposure to mortality as measured by sibling deaths in childhood. We use exposure to community mortality and sibling deaths to assess more fully if there is a causal link between early-life mortality exposure and subsequent adult mortality between age 18 and 85. Furthermore, we assess the age-specific influences of mortality exposure and follow individuals through the life course. Early- and midlife characteristics such as socioeconomic status are included to control for mediating factors. We assess the effect of mortality exposure on all-cause and cause-specific mortality to better reveal possible causal mechanisms. Furthermore, we examine the role of mortality selection and investigate which exposure ages in childhood are most strongly associated with adult mortality. Finally, we compare two populations to assess the effect of exposure to mortality in different mortality régimes.

## Data and Methodology

We use two unique, large-scale historical demographic datasets arising from two widely differing environments: the Utah Population Database (UPDB) and LINKS Zeeland, The Netherlands. Both data sources contain large-scale demographic information about historical populations with multigenerational pedigrees, which have been linked to spatial information—census enumeration districts and municipalities, respectively—which allow us to follow individuals and their siblings, parents and children over their life course. For Utah, we use birth cohorts 1874–1929, following individuals until 1959–2015, and for The Netherlands birth cohorts 1811–1872, following individuals until 1896–1957.

Utah, a western state in the USA, was first settled by immigrants from North America and Europe after 1847. Initially, many migrants were associated with The Church of Jesus Christ of Latter-day Saints (i.e., Mormon church), but over time the share of non-Mormons and inactive Mormons has grown. Despite poverty and harsh conditions in the early years of settlement, the population in Utah was relatively healthy (Mineau et al. 2004) in comparison with Zeeland, with lower infant and child mortality and higher life expectancy. Zeeland is a largely Protestant province in the southwestern corner of The Netherlands and consisted of relatively isolated islands. In Zeeland, a lack of potable water existed due to salinity. Adult life expectancy was low and infant and child mortality high, reaching 50% in some years and communities (Hoogerhuis 2003; Van Dijk and Mandemakers 2018). For both populations, high-quality family reconstitution databases exist: the Utah Population Database (UPDB), containing life courses of the population of Utah, and LINKS (Linking System for historical family reconstruction) Zeeland, containing life histories based on linked civil certificates. Both databases contain large numbers of individuals and multigenerational pedigrees with vital information linked to spatial information. These features make them highly suitable for assessing both contextual and familial effects on adult mortality. The UPDB has been used extensively to assess the effect of social, biological and genetic factors on longevity and lifespans (Garibotti et al. 2006; Kerber et al. 2001; Smith et al. 2009).

A portion of the UPDB is based on family group sheets on which genealogies of Utah founders and their descendants are recorded. These have been linked into intergenerational pedigrees with information from other official administrative and medical sources, including birth and death certificates and US Census records. The full database includes over 11 million individuals. Both individuals with and without an affiliation with The Church of Jesus Christ of Latter-day Saints (LDS or Mormons) are included in UPDB. For our analyses, we select individuals born in Utah between 1874 and 1929 for whom information is available about their parents and their own life course, and who have at least one sibling. Furthermore, eligible individuals must have survived to at least age 18 and are followed until death, out-migration, lost to follow-up or survival to age 85. There are 348,964 eligible individuals in the UPDB (see Table 1) whom we call egos and individuals interchangeably.

**Table 1:** Descriptive statistics

	Utah (UDPB)				Zeeland (LINKS)			
	Min	Max	Mean men	Mean women	Min	Max	Mean men	Mean women
Age at last observation	18.01	85.00	67.40	69.65	18.01	85.00	62.69	62.72
Observed until death	0	1	.72	.55	0	1	.88	.88
Cause-specific deaths (ICD)	0	1	.62	.68	–	–	–	–
Cardiovascular disease	0	1	.32	.33	–	–	–	–
Cancer	0	1	.12	.15	–	–	–	–
Respiratory disease	0	1	.07	.06	–	–	–	–
Digestive system	0	1	.03	.04	–	–	–	–
Nervous system	0	1	.03	.04	–	–	–	–
Metabolic disease	0	1	.02	.03	–	–	–	–
Genito-urinal system	0	1	.02	.02	–	–	–	–
Infectious disease	0	1	.01	.02	–	–	–	–
Community mortality < 1	.20	3.30	1.06	1.06	.54	5.11	2.58	2.60
District mortality 1–4	.04	2.35	.44	.44	.00	2.92	1.05	1.06
District mortality 5–18	.12	4.00	.56	.56	.00	2.41	.49	.49
District mortality 0–18	.66	5.71	1.93	1.92	1.22	8.30	4.13	4.15
Number of siblings	1	20	6.01	6.06	1	24	7.53	7.53
Sibling deaths								
Ego 0–18	0	12	.52	.50	0	13	1.81	1.82
Ego 0–5	0	7	.17	.17	0	7	.78	.79
Ego 6–18	0	8	.34	.34	0	10	1.03	1.03
Ego unexposed	0	16	.40	.41	0	16	.97	1.01
Ego in utero	0	5	.02	.02	0	4	.06	.06
Ego 0–18, to infection	0	4	.04	.04	–	–	–	–
Birth interval < 18 months	0	1	.20	.20	0	1	.32	.32
Birth order	1	20	4.04	4.06	1	24	4.28	4.32
Twin birth	0	1	.02	.02	0	1	.01	.01
Age mother at birth								
12–19	0	1	.06	.06	0	1	.01	.01
20–29	0	1	.53	.53	0	1	.48	.48
30–34	0	1	.21	.21	0	1	.27	.27
35–50	0	1	.21	.21	0	1	.24	.24
Mother dead < 45	0	1	.07	.08	0	1	.13	.13
Father dead < mother 45	0	1	.08	.08	0	1	.12	.13
SES father	0	100	30	31	41	99	51	51
Father is a farmer	0	1	.38	.38	0	1	.21	.20
SES father missing	0	1	.02	.03	0	1	.00	.00
SES	0	100	40	41	41	99	51	52
Farmer	0	1	.22	.19	0	1	.17	.15
SES missing	0	1	.23	.29	0	1	.09	.20
Inactive mormon	0	1	.30	.28	–	–	–	–
Active mormon	0	1	.48	.52	–	–	–	–
Share men/women			.51	.49			.49	.51
Total number men/women			178,404	170,560			39,849	41,022

LINKS is based on digitized vital event registration certificates that identify the individual and his or her parents. These have been used to reconstruct life courses by linking certificates pertaining to the same individuals using first and last names of the ego and his or her parents, spouses and children. The full database contains information on almost 2 million individuals from Zeeland who experienced a vital event in that province between 1812 and 1912 for births, 1812 and 1927 for marriages, and 1812 and 1957 for deaths. For the analysis, we select 80,871 persons with a known date of birth and death, as life course reconstructions have been shown to be reliable for these cases (Van den Berg et al. 2018), who were born between 1811 and 1872, whose parents are known and who have at least one sibling (see Table 1). Individuals are followed from age 18 until death or censoring at age 85.

In this paper, we examine mortality exposure in different phases of childhood. We consider exposure to sibling deaths during three phases: (1) from conception until birth, (2) birth until the sixth birthday and (3) between the sixth and the 18th birthday. Additionally, we include sibling deaths before subjects are conceived, as these may still influence ego’s adult mortality. Families may fail to provide children with adequate nutrition or care, leading to both increased childhood mortality and adult mortality among all offspring. Thus, it is suggested that the causal mechanism works broadly through adverse early-life conditions in the family rather than through direct exposure to sibling deaths.

1. Measuring Community-Level Mortality

To assess exposure to infant and child mortality at the contextual level, we use two related approaches for Utah and The Netherlands. For Utah, we calculate infant and early and late child mortality rates for 10-year periods based on decennial census information aggregated into census enumeration districts (i.e., these approximate neighborhoods and represent a space that can be navigated by a census enumerator). For The Netherlands, we calculate infant, early and late child mortality rates and total child mortality rates for municipalities by year. For both populations, community-level mortality rates are per 1000 children. Variables are centered at the grand mean for their respective populations.

In Utah, birth certificates were not implemented before 1904. As the US decennial Census records for Utah are linked to the UPDB starting in 1880, we identify parents and parental enumeration districts and find their children through the UPDB. For instance, children born between 1905 and 1915 were linked to their parental enumeration districts in the year 1910. For the years 1874–1890, 1890–1905, 1905–1915, 1915–1925 and 1925–1929, the censuses for the years 1880, 1900, 1910, 1920 and 1930 are used, respectively. Unfortunately, nearly the entire US census for 1890 was destroyed in a fire. Based on the census records and UPDB, we calculate the total number of children born and the percentage of deaths before the age of 1, ages 1–4 and 5–17 in each census enumeration district for ten-year periods. For Zeeland, we calculate comparable rates based on indexes of birth and death certificates per municipality per year. Mortality rates are averaged over five-year periods after birth, as there are small numbers of births and deaths in some municipalities. We use the municipality and year of birth to link individuals to the appropriate area-level mortality levels. Stillbirths are not included.

2. Sibling Mortality

Sibling deaths are measured separately for different ages of ego. We include the total number of deaths among full siblings of ego, regardless of the sibling’s age, between ego’s birth and age six; between ego’s age six and 18, and total number of sibling deaths between birth and eighteenth birthday. Furthermore, we consider sibling deaths to which ego was not exposed, occurring before ego’s conception, and deaths occurring while ego was in utero. For Utah, we examine the number of sibling deaths from infectious disease (in parallel with non-infectious deaths) in 1904 or later, when death certificates with causes of death are available. In all analyses, we control for the number of siblings ever born.

3. Additional Covariates

We control for several characteristics of the family of origin and circumstances in adulthood. Descriptive statistics can be found in Table 1. Demographic characteristics of individuals include the number of full siblings, birth order, age of the mother at birth, and birth interval to prior birth. For Utah, we include an indicator of religious status. Active Mormons generally engage in lifestyle characteristics beneficial for survival, such as alcohol and tobacco avoidance, fasting, and participating in social aspects of the religion. Inactive LDS members were baptized in the LDS church, generally at age eight, and share an early-life environment which may be beneficial for survival, but did not express their commitment to the LDS church in adulthood. Non-LDS members have no record of involvement with the LDS church. No individual-level indicator for denomination was available for the predominantly Protestant Zeeland.

With regard to midlife characteristics, we include deaths of ego’s parents before the mother reaches the end of her reproductive period (age 45), as the numbers of ego’s siblings and deaths in the sibship are affected by survival of the parents. We also include the highest socioeconomic status (SES) of the father and the individual and, given the large fraction of the populations engaged in agriculture, we distinguish farmers from non-farmers as a dummy variable. For Utah, we use the Nam-Power SES score (1950 version) (Nam and Boyd 2004), using the highest SES observed in the censuses and, if unavailable, SES from the death certificate of the father. For Zeeland, we use the highest HISCAM SES (Lambert et al. 2013) from any linked vital event certificate to measure socioeconomic status, and HISCLASS (Van Leeuwen and Maas 2011) to distinguish farmers from non-farmers. For persons with a missing SES score, we impute the mean score and include a dummy indicating whether the SES score was imputed. For this period, males were the primary breadwinner for both Utah and Zeeland, resulting in under-registration of female labor participation with few recorded occupations for women. Therefore, for women, we use the socioeconomic status of the spouse. If there is no known spouse (Utah 13.7%, Zeeland 19.8%), we assign the mean male socioeconomic status and a dummy variable indicating whether missing values were imputed and included as a covariate.

## Results

We estimate sex-specific Cox proportional hazard regressions. Individuals are followed from age 18 until death, last date of observation (Utah) or censoring at age 85, the oldest achievable age for the last Utah birth cohort (1929–2015). All models are estimated with R version 3.3.2. Men have higher mortality rates than women for sociocultural and biological reasons, including behavioral patterns, the protective role of estrogen, suppressing effect of testosterone on immunity, and an unprotected X chromosome (Lindahl-Jacobsen et al. 2013). The effect of early-life conditions on survival may therefore be stronger for men than women (Doblhammer et al. 2013; Smith et al. 2009); on the other hand, cohort mortality may affect both women and men similarly (Lindeboom et al. 2010). We analyze the relative risk of mortality first by exposure to high infant, early and late childhood mortality in the community and second by exposure to sibling mortality in the first 6 and 18 years of life. Finally, we analyze the cause-specific mortality risks using a competing risk approach. We first present crude, unadjusted effects in Model 1. In Model 2, we add demographic characteristics of the family to control for possible confounders. In Model 3, we assess whether effects are mediated by a set of midlife characteristics. All analyses are stratified by year of birth to account for temporal changes in exposure to sibling mortality and adult mortality hazards. In these stratified models, the hazard of adult mortality as a function of exposure to sibling mortality is estimated based on comparisons to individuals from the same birth cohort.

1. Exposure to Mortality in the Community

We begin by reporting results that demonstrate crude (unadjusted) associations between exposure to community mortality during childhood and adult mortality past age 18. For Utah, we use all egos who can be linked to a parental enumeration district closest to their birth: 149,285 men and 141,992 women. For Zeeland, we use places of birth and municipal mortality rates in the first five years after birth. Estimates of relative risks are based on a community-level increase of mortality by 10%. Results are shown in Table 2.

**Table 2:** Childhood community levels of mortality among infants, young and older children and survival between age 18 and 85 in Utah and Zeeland

District mortality	Model 1				Model 2				Model 3			
	Utah		Zeeland		Utah		Zeeland		Utah		Zeeland	
	HR	95% CI	HR	95% CI	HR	95% CI	HR	95% CI	HR	95% CI	HR	95% CI
Men												
Infants	**1.09**	1.07–1.12	**1.01**	1.00–1.03	**1.08**	1.06–1.11	**1.02**	1.00–1.04	1.02	1.01–1.06	1.01	0.99–1.03
Young children	**1.10**	1.05–1.14	**1.07**	1.03–1.11	**1.08**	1.02–1.10	**1.06**	1.02–1.10	0.99	0.95–1.03	**1.04**	1.00–1.08
Older children	**1.17**	1.13–1.21	**1.09**	1.04–1.15	**1.15**	1.08–1.16	**1.07**	1.02–1.12	1.03	0.99–1.06	**1.06**	1.01–1.11
All mortality	**1.08**	1.07–1.10	**1.03**	1.01–1.04	**1.07**	1.05–1.08	**1.03**	1.01–1.04	1.01	1.00–1.03	**1.02**	1.00–1.03
N	149,285		39,849									
Events	106,568		35,404									
Women												
Infants	**1.08**	1.05–1.12	**1.05**	1.03–1.07	**1.08**	1.05–1.11	**1.06**	1.04–1.07	**1.03**	1.02–1.08	**1.06**	1.04–1.08
Young children	**1.13**	1.08–1.18	**1.03**	1.00–1.07	**1.12**	1.06–1.16	**1.03**	1.00–1.07	**1.05**	1.01–1.11	1.01	0.98–1.05
Older children	**1.09**	1.05–1.14	1.00	0.96–1.05	**1.08**	1.02–1.10	1.00	0.96–1.05	0.99	0.96–1.04	0.96	0.92–1.01
All mortality	**1.07**	1.05–1.09	**1.04**	1.02–1.05	**1.07**	1.04–1.08	**1.04**	1.02–1.05	1.02	1.01–1.05	**1.03**	1.03–1.05
N	141,992		41,022									
Events	77,449		36,289									

All models are stratified by birth year. Bold: significant effects *p* < 0.05

Model 1: uncontrolled effects. Model 2: controlled for number of siblings, birth interval, birth order, age mother at birth and twin birth and socioeconomic characteristics of the family of origin. Model 3: controls Model 2 plus survival of father and mother until end of the reproductive period of the mother, religion (Utah) and socioeconomic characteristics in adulthood

Estimates of the relation between community mortality rates in childhood and adult mortality rates show that in Utah and Zeeland, there is a significant positive relation between community mortality rates for infants, young and older children and adult mortality rates for men and women. For females in Zeeland, only infant mortality, early child mortality and total child mortality are related to adult mortality risk (Table 2, Model 1). After including controls for demographic characteristics of the individual and the family of origin and socioeconomic characteristics of the family of origin, these effects remain significant. In Model 3, we add individual-level control variables including SES, survival of parents and religious status (Utah). For men in Utah, we no longer find a significant association between childhood mortality rates in the community and mortality rates in adulthood. Most of the effects are mediated by religion in adulthood (results not shown). For Utah women, effect sizes are reduced but effects remain significant for infant mortality rates and early child mortality rates. For Zeeland, early child mortality is no longer significantly associated with male adult mortality rates and infant mortality is no longer significantly associated with female adult mortality rates. For women, we find that infant and total child mortality affect adult mortality rates for Zeeland. Overall, for both Utah and Zeeland, the most consistent effects of community-level mortality when egos were children were infant mortality for women. For Zeeland, we also find an effect of all childhood mortality in their community for both males and females.

2. Exposure to Sibling Mortality

A proximate measure of exposure to death, most likely from infectious diseases, is childhood deaths among ego’s siblings. In Table 3, results can be found for Cox proportional hazard models analyzing the mortality hazard rate between ages 18 and 85. Proportionality tests do not indicate that the effects of the covariates vary with age for either men or women in Zeeland or Utah.

**Table 3:** Sibling deaths in childhood and survival between age 18 and 85

Sibling deaths		Model 1				Model 2				Model 3			
		Utah		Zeeland		Utah		Zeeland		Utah		Zeeland	
		HR	95% CI	HR	95% CI	HR	95% CI	HR	95% CI	HR	95% CI	HR	95% CI
*Men*													
Ego unexposed	1 or more deaths	**1.04**	1.02–1.05	**1.02**	1.00–1.05	**1.04**	1.02–1.05	1.02	0.99–1.05	**1.04**	1.02–1.05	1.02	0.99–1.04
Ego in utero	1 or more deaths	**1.05**	1.00–1.10	**1.05**	1.01–1.10	**1.05**	1.00–1.10	**1.05**	1.00–1.10	1.04	0.99–1.08	1.04	0.99–1.09
Ego 0–5	1 death	**1.05**	1.03–1.06	**1.03**	1.01–1.06	**1.04**	1.03–1.06	**1.03**	1.01–1.06	**1.04**	1.02–1.06	**1.03**	1.00–1.05
	2 deaths	**1.08**	1.03–1.12	**1.10**	1.06–1.13	**1.07**	1.03–1.11	**1.09**	1.06–1.13	**1.06**	1.02–1.10	**1.08**	1.05–1.12
	More than 2 deaths	1.08	0.98–1.18	**1.08**	1.03–1.13	1.07	0.98–1.17	**1.08**	1.03–1.13	1.07	0.97–1.17	**1.07**	1.02–1.12
Ego 6–18	1 death	**1.07**	1.06–1.09	1.02	0.99–1.05	**1.08**	1.06–1.09	**1.04**	1.01–1.06	**1.07**	1.05–1.08	**1.03**	1.01–1.06
	2 deaths	**1.08**	1.05–1.10	**1.07**	1.03–1.11	**1.08**	1.06–1.11	**1.10**	1.06–1.13	**1.07**	1.05–1.10	**1.09**	1.05–1.13
	More than 2 deaths	**1.11**	1.06–1.15	**1.07**	1.04–1.11	**1.11**	1.07–1.16	**1.11**	1.07–1.16	**1.10**	1.05–1.14	**1.10**	1.06–1.15
Ego 0–18	1 death	**1.08**	1.06–1.09	1.02	0.99–1.05	**1.08**	1.06–1.09	1.02	0.99–1.05	**1.07**	1.06–1.09	1.02	0.99–1.05
	2 deaths	**1.09**	1.07–1.12	**1.08**	1.05–1.12	**1.09**	1.07–1.12	**1.09**	1.06–1.13	**1.08**	1.06–1.11	**1.08**	1.05–1.12
	More than 2 deaths	**1.12**	1.09–1.15	**1.11**	1.07–1.15	**1.12**	1.09–1.16	**1.14**	1.10–1.18	**1.11**	1.08–1.14	**1.13**	1.09–1.17
	Events	127,749		35,404									
	N	178,404		39,849									
Ego 0–18	Infection, 1 or more deaths	**1.06**	1.02–1.10			**1.06**	1.02–1.10			**1.04**	1.00–1.08		
	Events	67,112											
	N	99,800											
*Women*													
Ego unexposed	1 or more deaths	**1.04**	1.03–1.06	**1.04**	1.02–1.06	**1.04**	1.02–1.06	**1.03**	1.00–1.05	**1.04**	1.02–1.06	**1.03**	1.01–1.06
Ego in utero	1 or more deaths	**1.04**	1.00–1.08	0.99	0.95–1.04	1.03	0.98–1.08	0.99	0.94–1.03	1.03	0.97–1.08	0.99	0.94–1.03
Ego 0–5	1 death	**1.12**	1.10–1.17	**1.03**	1.00–1.05	**1.11**	1.09–1.13	**1.03**	1.01–1.06	**1.11**	1.09–1.14	**1.03**	1.00–1.05
	2 deaths	**1.12**	1.07–1.17	1.02	0.98–1.05	**1.11**	1.07–1.16	1.02	0.99–1.05	**1.11**	1.06–1.16	1.02	0.99–1.05
	More than 2 deaths	**1.16**	1.05–1.28	0.99	0.95–1.04	**1.15**	1.04–1.27	1.00	0.95–1.04	**1.18**	1.07–1.30	0.99	0.95–1.04
Ego 6–18	1 death	**1.10**	1.08–1.11	1.02	0.99–1.04	**1.10**	1.08–1.11	**1.03**	1.00–1.06	**1.10**	1.08–1.12	**1.04**	1.01–1.06
	2 deaths	**1.12**	1.08–1.15	**1.04**	1.00–1.07	**1.12**	1.09–1.15	**1.06**	1.03–1.10	**1.12**	1.08–1.15	**1.07**	1.04–1.11
	More than 2 deaths	**1.09**	1.04–1.14	**1.05**	1.01–1.09	**1.10**	1.05–1.15	**1.10**	1.05–1.14	**1.10**	1.05–1.15	**1.12**	1.08–1.17
Ego 0–18	1 death	**1.14**	1.12–1.16	**1.03**	1.00–1.06	**1.14**	1.12–1.15	**1.03**	1.00–1.06	**1.14**	1.12–1.16	**1.04**	1.01–1.07
	2 deaths	**1.15**	1.12–1.17	1.02	0.99–1.06	**1.15**	1.12–1.18	**1.04**	1.00–1.07	**1.15**	1.12–1.18	**1.04**	1.00–1.07
	More than 2 deaths	**1.16**	1.12–1.20	**1.05**	1.02–1.08	**1.16**	1.13–1.20	**1.08**	1.04–1.11	**1.16**	1.13–1.20	**1.09**	1.05–1.13
	Events	94,047		36,289									
	N	170,560		41,022									
Ego 0–18	Infection, 1 or more deaths	**1.13**	1.08–1.18			**1.13**	1.08–1.18			**1.12**	1.07–1.17		
	Events	46,701											
	N	95,083											

See Table 2. Model 1 controls for number of siblings

Results show that sibling deaths are related to higher relative risk of mortality for both men and women. In Utah, where relatively few sibling deaths occur, sibling mortality has a stronger effect on female mortality in adulthood than on male adult mortality. In Zeeland, where sibling deaths are relatively common, male adult mortality is particularly affected. Here, greater exposure in the family of origin—2 or more sibling deaths—is significantly and adversely associated with adult mortality, whereas in lower-mortality Utah one or two sibling deaths are also significantly related to higher mortality in adulthood. The effects of sibling mortality are not mediated by the demographic and socioeconomic characteristics of the family of origin or adulthood in either population. For both men and women, there is a dose response pattern that indicates that with increasing sibling deaths there is increased adult mortality for both populations (see Table 4), controlling for sibship size.

**Table 4:** Test of trend for exposure to sibling deaths in childhood and adult mortality age 18–85

	Men			Women		
	HR	95% CI	N events	HR	95% CI	N events
*Zeeland*						
All-cause mortality	**1.05**	1.01–1.04	35,404	**1.03**	1.04–1.06	36,289
*Utah*						
All-cause mortality	**1.05**	1.04–1.05	127,749	**1.07**	1.06–1.08	94,047
Cardiovascular	**1.05**	1.04–1.07	40,781	**1.07**	1.06–1.09	30,875
Cancer	**1.03**	1.01–1.06	15,174	**1.07**	1.05–1.10	13,736
Respiratory system	**1.07**	1.05–1.10	9305	**1.12**	1.08–1.16	5497
Digestive system	**1.09**	1.05–1.14	4297	**1.09**	1.04–1.14	3635
Nervous system	**1.06**	1.02–1.11	3444	**1.10**	1.06–1.15	3602
Metabolic disease	**1.07**	1.01–1.13	2532	**1.12**	1.07–1.17	3282
Genitourinary system	**1.07**	1.01–1.13	1980	1.05	0.99–1.12	1928
Infectious disease	1.04	0.99–1.11	1848	1.06	0.98–1.12	1481

Significant effects (*p* < 0.05) are shown in bold. Models are stratified by birth year and control for number of siblings, birth interval, birth order, age mother at birth and twin birth, socioeconomic characteristics of the family of origin, survival of father and mother until end of the reproductive period of the mother and religion (Utah)

Given that there is an effect of sibling deaths during the life of ego as well as before ego’s conception, it is likely that the causal force underlying the observed association is based on family circumstances. These circumstances include dietary choices, life style characteristics, stress experienced by parents after child loss, as well as direct exposure to infections and disease of siblings, features that are shared within families. For Zeeland women, exposure to deaths during late childhood appears more lethal than those occurring earlier in life, while in Utah both are related to adult survival. For men, sibling deaths in both early and late childhood are related to adult mortality. Possibly, for women, caretaking tasks in late childhood play a role. As older girls were expected to care for sick siblings, there may be stronger exposure to their siblings’ illnesses and deaths in this period of life. Alternatively, girls could be more susceptible to illness and disease in the teenage years, as research has found excess mortality among girls during this period of life (Devos 2006). In line with these explanations, we find that siblings’ deaths due to infectious disease affect female mortality in adulthood more than male mortality, although the difference between men and women is not significant (results not shown).

3. Causes of Death

**Fig. 1:**
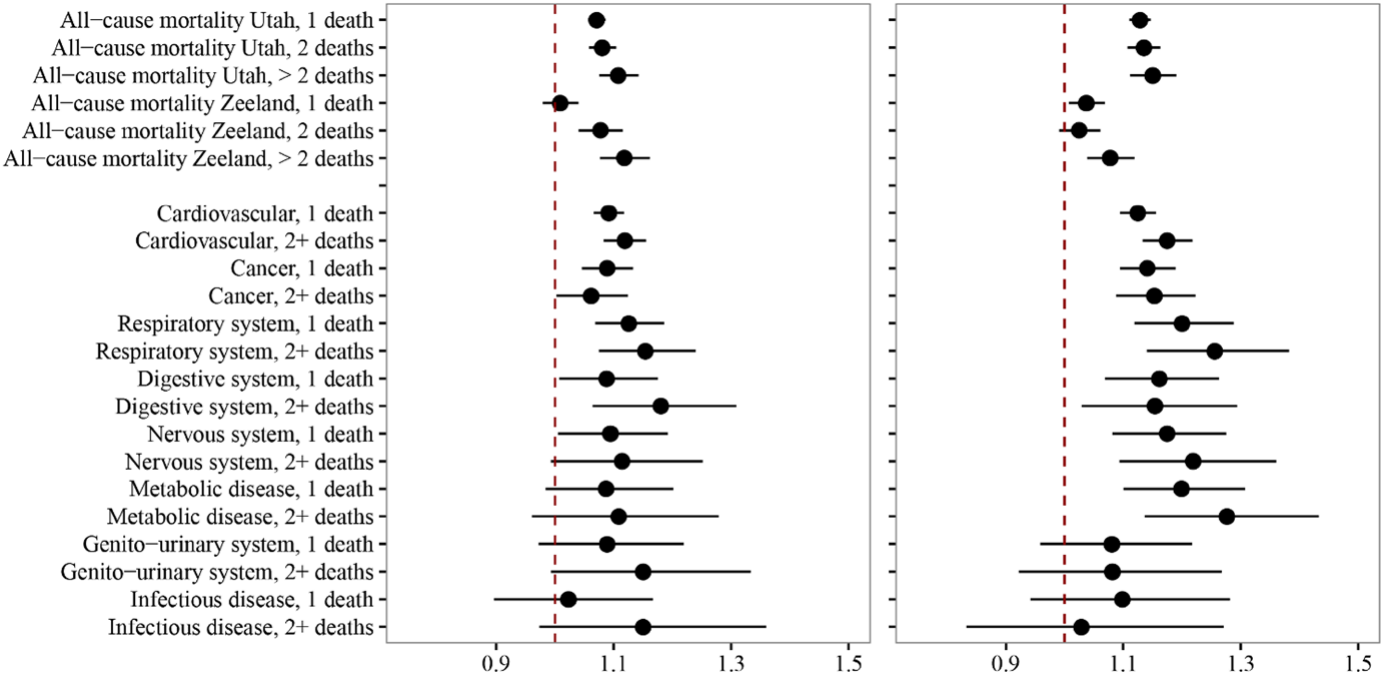
Causes of death by number of sibling deaths, men (left) and women (right). Notes Fig. 1: Relative risk, 95% confidence interval. Cause-specific mortality concerns Utah. Models are stratified by birth year. Models control for number of siblings, mother’s age at birth, birth order and interval, religion (Utah) and socioeconomic characteristics of the father

To investigate further the mechanism linking exposure to mortality in childhood and increased risks of adult mortality, we analyze the link between causes of death for ego and exposure to sibling mortality. Causes of death are not available for Zeeland. The causes of death have been collapsed into 17 broad categories adopted by the US National Center for Health Statistics using the International Classification of Diseases (ICD) codes. We include the following selected causes of death: infection, all cancers, cardiovascular disease, metabolic and endocrine systems, nervous system, respiratory system, digestive system and diseases of the genitourinary system. We apply a competing risk approach, estimating the risk of death before the age of 85 for each of these specific causes of death where we treat deaths from all other causes as censored when analyzing any specific cause of death. Descriptive statistics for the causes of death can be found in Table 1; results are summarized in Fig. 1.

Results show that there is a significant relation between exposure to mortality of siblings in the family of origin and deaths due to cardiovascular disease, cancers, diseases of the respiratory system and the digestive system. For women, the effect is also present for metabolic diseases and diseases of the nervous system (see Fig. 1). Tests of trend (see Table 4) show that there is a dose response pattern between the number of sibling deaths for men and women for all-cause mortality, cardiovascular disease, cancer, diseases of the respiratory system, digestive system, nervous system, genitourinary system (women) and metabolic diseases. In addition, sibling deaths due to infection are significantly related to ego all-cause mortality, and death of cardiovascular disease and metabolic disease for men and women, and to deaths of respiratory and digestive system diseases (see Table 5). A linear contrast between sibling deaths due to infectious disease and other causes of death indicates that all-cause mortality and mortality from cardiovascular disease and metabolic disease are affected differently by sibling deaths to infectious disease than by sibling all-cause mortality (see Table 5). That also applies to systems of the digestive system, but here, sibling deaths to infectious disease do not affect risk of death to diseases of the digestive system significantly.

**Table 5:** Cause-specific mortality age 18–85 by sibling death to infection, Utah

	Deaths to infectious disease		Other deaths		N events	Diff. deviance	p value
	HR	95% CI	HR	95% CI			
All-cause mortality	**1.08**	**1.05–1.11**	**1.16**	**1.14–1.17**	113,813	**17.46**	**.00**
Cardiovascular	**1.11**	**1.06–1.16**	**1.17**	**1.14–1.19**	35,286	**3.82**	**.05**
Cancer	1.01	0.94–1.09	**1.16**	**1.12–1.20**	18,319	1.86	.17
Respiratory system	**1.11**	**1.00–1.23**	**1.20**	**1.14–1.27**	8103	0.51	.47
Digestive system	1.12	0.97–1.30	**1.24**	**1.15–1.32**	4086	**4.49**	**.03**
Nervous system	**1.27**	**1.08–1.49**	**1.28**	**1.18–1.39**	3532	2.49	.11
Metabolic disease	**1.40**	**1.20–1.63**	**1.19**	**1.10–1.29**	3485	**3.78**	**.05**
Genitourinary system	1.10	0.83–1.32	**1.13**	**1.12–1.41**	1798	0.76	.38
Infectious disease	1.11	0.86–1.44	**1.22**	**1.07–1.39**	1314	1.36	.24

Total number of cases: 194,883 (men 99,800; women 95,083). Difference in deviance is based on nested models that estimate the effects of siblings who die from any cause first—treating the effects of sibling deaths from infectious and non-infectious deaths as having the same effect—compared to a model where sibling deaths due to infectious disease and other causes of death are allowed to have distinct effects

Models are stratified by birth year and sex, and control for number of siblings, birth interval, birth order, age mother at birth and twin birth, socioeconomic characteristics of the family of origin, survival of father and mother until end of the reproductive period of the mother and religion

Significant effects (*p* = .05) are shown in bold

4. Robustness Checks

We conducted several sensitivity analyses. First, we selected only cases where parents lived until the end of the reproductive period of the mother, as death of a parent likely affects sibling survival and adult mortality. We also selected only individuals with birth order two and up, as firstborn children may not be as likely to be exposed to sibling mortality and never had sibling mortality before their conception by definition. In addition, we conducted robustness checks estimating models with all effects included simultaneously, and using only individuals whose parents were located in the census records close to their year of birth. Results are available on request and were not substantively different from the analyses presented here.

## Conclusion and Discussion

We have examined whether exposure to deaths during childhood may create enduring health effects. The premise of our approach is that families play a pivotal role by constructing the circumstances by which children were exposed to mortality among their siblings. In line with work by Finch and Crimmins (Crimmins and Finch 2006; Finch and Crimmins 2004), we showed that there is an association between exposure to mortality in communities and birth cohorts and subsequent adult mortality rates. Especially for infant and all-child mortality, we detect effects on adult mortality rates, consistent with some research (Schellekens and van Poppel 2016) but only partially in line with findings by Finch and Crimmins (2004), who suggest that primarily infant mortality is related to adult mortality risk.

Research shows that exposure to sibling deaths is related to later-life stress (Norton et al. 2017) and increases mortality in early and late childhood and early adulthood (Yu et al. 2017). In line with these results, we found that sibling deaths are related to reduced survival at adult ages. Indeed, we found stronger evidence for negative long-term effects of exposure to sibling deaths than community-level exposure to mortality. After taking family-level characteristics and individual-level characteristics into account, we found that exposure to sibling deaths has enduring effects on adult mortality rates. Community-level mortality has a persistent effect on adult mortality as well, but effects are weaker, reduced after including family characteristics and became insignificant after including midlife characteristics.

We did not find evidence that weak mortality crises have a selection effect that overwhelms potential scarring effects. Putative scarring effects appeared to dominate selection effects, even if there is only a single exposure to sibling death (Hatton 2011). Despite the potential ameliorating effects of immunization and access to modern health technologies and care including antibiotics, we observed that even a single exposure to a sibling death in Utah is related to adult mortality rates. In Zeeland—where child mortality was very high—exposure to multiple sibling deaths is adversely related to adult survival, while exposure to only one sibling death is not. Possibly, in environments with high mortality the event of a sibling death does not necessarily contribute to adult mortality risks, as individuals are likely to be exposed to deaths in childhood in general. Considering lower adult life expectancies in Zeeland, the cumulative damage of adverse exposures is stronger in Zeeland than in Utah, an explanation for weaker effects of sibling deaths in Zeeland. In line with this explanation, we find that in Zeeland and Utah effects of sibling mortality are stronger in later, lower-mortality birth cohorts (results available on request).

In contrast to work which found no link between exposure to infectious disease and all-cause mortality, deaths of cardiovascular disease, cancer and stroke (Hayward et al. 2016), we found that exposure to sibling deaths is related to all-cause adult mortality as well as mortality due to cardiovascular disease, cancer, diseases of the respiratory, digestive and nervous system (women), and metabolic disease (women). Thus, exposure to all-cause early-life mortality among siblings is related to an array of causes of death and appears to be a general risk factor for premature adult mortality. In addition, the mortality risk of these diseases follows a dose response pattern. The extent to which individuals are exposed to sibling mortality is important for understanding adult survival: the greater the number of sibling deaths, the stronger the effect on adult all-cause mortality and cause-specific mortality. On the other hand, exposure to sibling deaths due to infection is related to cardiovascular disease and metabolic diseases for men and women. Thus, deaths attributable to infections appeared to be specifically related to chronic inflammatory responses. Sibling all-cause mortality is related to several common causes of death, suggesting that there may be a mechanism beyond inflammatory immune responses at play relating early-life exposure to mortality to adult mortality rates.

A few limitations are noteworthy regarding the study design. Measuring infant and early and late childhood mortality constituted a challenge both for the Utah and Zeeland data. For the Netherlands, out-migration information is unknown, and therefore, we used death risks relative to birth rates. For Utah, we necessarily relied on census data, which requires us to accept 10 years between measurements of several key variables. As a result, mortality conditions for individuals and families are measured at different ages of the egos and estimates of the relation between mortality exposure and adult mortality are conservative. Furthermore, while we were able to distinguish exposure to sibling deaths at several ages of the ego, we included mortality among siblings at all ages, while mortality among siblings of comparable age could be more influential than deaths among siblings who differ in age. Finally, although we were better able than existing research to distinguish between individuals who were most likely and less likely to have been exposed to illness and disease, it remains unknown whether individuals fell ill themselves. Using modern data, it should be possible to determine the extent to which earlier illnesses are related to lower adult life expectancies.

Frailty (random effects) models constitute an alternative modeling approach to the family-clustered survival models that were employed in this study. These models have the advantage of comparing adult mortality between exposed and unexposed individuals within families, thus controlling for family-unobserved heterogeneity. These models rely on a strict assumption that family-specific frailty is independent of the other exposures, notably sibling deaths. It is unlikely that frailty, as an indicator of family-specific risk of death, is independent of exposure to sibling deaths. Moreover, it is likely that exposure to sibling mortality is a sibling-shared experience because of the included age ranges of egos in relation to ages of their siblings. Alternatively, to estimate family-fixed effect models a much more select sample would have been selected for analysis, as at least three siblings need to be observed: two siblings that survive into adulthood and a third sibling to put them at risk of sibling mortality in childhood. This restriction would have resulted in a much smaller sample size (a 40 and 30% reduction of sample size in Utah and Zeeland, respectively). We therefore included age-specific exposure to sibling deaths in our main models rather than using frailty models and assessed the effects of family-shared characteristics by incorporating the effect of sibling deaths that occur before the birth of the ego. Results show that at least part of the effect of sibling mortality on adult mortality works at the family level rather than the individual level.

The strengths of this paper are first that we have been able to show convincingly the enduring effects of sibling deaths on adult mortality risk over the life course. Secondly, we have used two large high-quality databases on historic populations from Zeeland and Utah, with follow-up until 2015 for Utah, which have enabled us to compare across mortality régimes and living conditions. This implies that the relationship that we identified between mortality exposure and adult mortality risk is applicable to populations across contexts. Further, the results that we found were subjected to several robustness checks, which adds to the strength of our analysis. We were able to assess mechanisms linking early-life mortality exposure to adult mortality risk by studying micro-level causes of death. We jointly assessed community and family effects. Although research has suggested that period influences are more important than early-life exposures (Myrskylä 2010), we have been able to show that early-life exposures do matter greatly for adult health and mortality and that the family plays an important role in shaping and mediating these influences.
